# On Displacement of the Lower Fragment in Fracture of the Surgical Neck of the Humerus

**Published:** 1846-11

**Authors:** 


*On displacement of the lower fragment in fracture of the surgical neck of the Humerus.* By M. Debrou.—As fracture of the surgical neck of the humerus is commonly occasioned by direct violence acting on the posterior part of the shoulder or on the upper and outer part of the arm. M. Debrou thinks that the fracture is usually directed obliquely from above downwards, and from within outwards, or from before backwards. In 1843 he saw three cases of this fracture at the Hôtel Dieu of Orleans in which the obliquity was in this direction, and in those three cases there was considerable prominence of the lower fragment, which was drawn upwards and carried inwards and forwards. When the extremity of the lower fragment is drawn in this direction it encounters a smaller thickness of soft parts than if it were displaced externally, and may come in immediate contact with or even run the risk of perforating the skin. In the three cases above referred to, the extremity of the bone partially (but not completely) perforated the skin, and thence carried the integument backwards with it on the slightest motion of the elbow and of the inferior fragment; from this there resulted a depression of the skin, which became deeper the further the lower fragment was carried backward. In one of these cases the lower fragment was so firmly engaged in the skin that perfect reduction could not be effected until the bone was freed from the skin by means of a subcutaneous section with a tenotome introduced two inches from the site of the fracture.—*Dub. Med. Press, from Jour. de Chirurgie.*

